# *BEM2*, a RHO GTPase Activating Protein That Regulates Morphogenesis in *S. cerevisiae*, Is a Downstream Effector of Fungicidal Action of Fludioxonil

**DOI:** 10.3390/jof8070754

**Published:** 2022-07-21

**Authors:** Anupam Sharma, Yogita Martoliya, Alok K. Mondal

**Affiliations:** 1 CSIR-Institute of Microbial Technology, Sector 39A, Chandigarh 160036, India; anu16jl@gmail.com; 2 School of Life Sciences, Jawaharlal Nehru University, New Delhi 110067, India; yogitamartoliya12@gmail.com

**Keywords:** antifungal agent, fludioxonil, hybrid histidine kinase, cell wall integrity pathway, GTPase activating protein (GAP)

## Abstract

Fludioxonil belongs to the phenylpyrrole group of fungicides with a broad antifungal spectrum that has been widely used in agricultural practices for the past thirty years. Although fludioxonil is known to exert its fungicidal action through group III hybrid histidine kinases, the downstream effector of its cytotoxicity is poorly understood. In this study, we utilized a *S. cerevisiae* model to decipher the cytotoxic effect of fludioxonil. Through genome wide transposon mutagenesis, we have identified Bem2, a Rho GTPase activating protein, which is involved in this process. The deletion of *BEM2* resulted in fludioxonil resistance. Our results showed that both the GAP and morphogenesis checkpoint activities of Bem2 were important for this. We also provided the genetic evidence that the role of Bem2 in the cell wall integrity (CWI) pathway and cell cycle regulation could contribute to the fludioxonil resistance phenotype.

## 1. Introduction

Fludioxonil belongs to the phenylpyrrole group of fungicides with a broad antifungal spectrum. It is used to control a variety of plant-pathogenic fungi [1,2,3,4]. Previous studies have revealed that fludioxonil acts as a fungicide through the inhibition of Nik1 orthologs or Group III hybrid histidine kinase (HHK3), which leads to the improper activation of the p38/HOG MAPK pathway in fungal pathogens [5,6,7,8,9]. Recent genetic studies on HHK3 orthologs have revealed that the null mutants are resistant to phenylpyrrole classes of fungicides [7,8], indicating that they could be the direct target of these fungicides or a signaling mediator for its fungicidal action that activates the p38/HOG MAPK pathway. The fungicidal action of fludioxonil causes the constitutive activation of p38/HOG MAPK pathways in fungi. This affects the cell in various ways, e.g., hyphal swelling and bursting at tips, metabolite leakage, alterations in cell wall synthesis, and defects in spore germination [10]. *S. cerevisiae* exhibits a natural resistance towards phenylpyrrole classes of fungicides. However, the heterologous expression of HHK3 orthologs from *Magnaporthe grisea*, *Alternaria brassicicola*, *Candida albicans*, *Candida lusitaniae*, or *Debaryomyces hansenii* confers a sensitivity towards these drugs to *S. cerevisiae* cells that indicates that the fungicidal effect of these compounds is mediated through HHK3 orthologs [7,11,12,13,14]. Recent studies carried out with *DRK1*, a HHK3 ortholog from *Blastomyces dermatitidis*, indicate that fludioxonil does not bind to Drk1 [4]. However, it increases the intracellular level of methylglyoxal by inhibiting the enzyme triosephosphate isomerase. The accumulation of methylglyoxal causes covalent thiol modification of Drk1, which becomes cytotoxic to the cells resulting in the inappropriate activation of the p38/HOG MAPK pathway [4]. However, the details of the molecular events leading to the subsequent growth arrest or cell death remained unclear. A recent study has implicated cellular processes such as vesicular trafficking, endocytosis, vacuolar functions, and cytokinesis, in the cytotoxic effect of fludioxonil [14]. It is possible that fludioxonil could act through unknown cellular targets to regulate these cellular processes.

In this study, we, therefore, carried out a transposon-based approach to identify the signaling pathways or components controlling the fludioxonil sensitivity using *S. cerevisiae* model and found Bem2, a known Rho GAP (GTPase activating proteins), to be involved in the antifungal action of fludioxonil. The deletion of *BEM2* resulted in fludioxonil resistance. We provide the genetic evidence that the role of Bem2 in the cell wall integrity (CWI) pathway and cell cycle regulation could contribute to the fludioxonil resistance phenotype.

## 2. Materials and Methods

### 2.1. Strains and Growth Conditions

Different deletion mutants used in this study were either procured from EUROSCARF or made in *S. cerevisiae* strains BY4742 or BY4741 background. YPD (1% yeast extract, 2% peptone, and 2% dextrose) and SD minimal medium (2% glucose and 0.67% yeast nitrogen base without amino acids) (Difco) with only required supplements were used for growing *S. cerevisiae* strains. Strains used in this study are listed in Supplementary Table S1. The transformation of *S. cerevisiae* strains with different plasmids was done by lithium acetate method [15]. Plasmids used in this study are listed in Supplementary Table S2.

### 2.2. Screening of S. cerevisiae Strain BY4742 with mTn3-LacZ/LEU2 Mutagenized Library

We obtained mTn3-LacZ/LEU2 mutagenized library [16] from the Yale Genome Analysis Center, New Haven, CT (http://ygac.med.yale.edu/mtn/reagent/avail_reagents/lacZLEU2_lib_p.stm accessed on 20 August 2010). Plasmid DNA from the pools of the library was digested with NotI and the linear DNA corresponding to the 8 kb region was purified and transformed into *S. cerevisiae* strain BY4742 harbouring pClNik1 plasmid using lithium acetate protocol. Transformants were selected on SD-Leu medium. After 24 h of incubation, the plates were replica plated on SD media containing fludioxonil (50 µg/mL) and incubated further for 2–3 days at 30 °C. Colonies that appeared on these plates were designated as fludioxonil resistant mutants. To identify the dominant resistant mutant in this pool, each resistant mutant was crossed with *S. cerevisiae* strain BY4741/pClNik1. The resulting diploids that grew on SD media containing fludioxonil (50 µg/mL) were considered as dominant resistant mutant. To rule out whether the appearance of fludioxonil resistance was due to the transposon insertion in *HOG1* or *PBS2,* osmo-sensitivity of all the resistant mutants was checked on plates containing 0.7 M NaCl. For transposon insertion in *SSK1* gene each recessive fludioxonil-resistant mutant was crossed with *Δssk1* (YPDahl143) harbouring pClNik1 and fludioxonil sensitivity of the resulting diploids was checked on SD media containing fludioxonil (50 µg/mL). Transposon mutants corresponding to those diploids still resistant to fludioxonil were discarded. To identify the transposon insertion site, genomic DNA immediately adjacent to the inserted *lacZ* sequences was obtained by a plasmid rescue method using pRSQ–*URA3* plasmid (GenBank U64694). Positive plasmids were sequenced using primer LacZ R having the binding site within the *lacZ* sequences of the terminal repeats. The nucleotide sequences were compared with the GenBank database using the BLASTN program.

### 2.3. Construction of BEM2 Mutants

The coding sequence of *BEM2* along with the upstream promoter region (7.1 kb) was amplified in two parts using BY4742 genomic DNA as a template. The first PCR fragment (~4.3 kb) was amplified by primers Bem2 PF and Bem2 Kas R, and the second fragment (~2.8 kb) was amplified by primers Bem2 Kas F and Bem2 ORF R (Table 1). The second PCR fragment (~2.8 kb) was cloned in the plasmid vector pGEM7z (Promega, Madison, WI, USA) digested by Sac1 restriction enzyme and treated with Klenow for blunt-end ligation. Positive clone with the desired orientation of the insert was selected by restriction enzyme digestion and was further used to clone the first fragment of (~4.3 kb) at Xho1/Kas1 sites. The resultant plasmid pGEM7z-BEM2 was confirmed by restriction digestion and DNA sequencing. Full-length *BEM2* ORF along with its promoter was then excised and subcloned in pRS313 [17] vector at Xho1/Sac1 sites to obtain pBEM2-313. The transformants were screened for the presence of insert by restriction digestion and confirmed by DNA sequencing. R2003A mutant was generated by replacing arginine with alanine residue at 2003 position in the GAP domain by site-directed mutagenesis using overlap extension PCR. First PCR was done to amplify ~1.4 kb fragment using Bem2 Sph1 F and R2003A R primers. Second PCR was done to amplify ~504 bp fragment using R2003A F and Bem2 ORF R primers. These PCR products were used as the template for the final overlap extension PCR to amplify ~1.8 kb fragment using Bem2 Sph1 F and Bem2 ORF R primers. ~1.8 kb PCR fragment was digested with Sph1 and Sac1 restriction enzymes and then cloned in pBEM2-313 at Sph1/Sac1 sites replacing the original fragment to obtain pBEM2-GAP. Similarly, Δ2-1749 mutant was made by overlap extension PCR using primer pairs, Bem2 PF/Δ2-1749R primers and Δ2-1749 F/Bem2 ORF R. The resultant plasmid pBEM2-1749 was confirmed by restriction digestion and automated DNA sequencing.

To construct double deletion mutants, *BEM2* was disrupted in *S. cerevisiae Δfks2*, *Δbni1*, *Δbnr1*, *Δskn7*, *Δcrz1*, *Δmbp1*, *Δswi6*, and *Δswe1* procured from EUROSCARF. The disruption cassette with *URA3* (1.7 kb) or *LEU2* marker (2.5 kb) genes was amplified from plasmid pUG72 or pUG73 [18], respectively, using primers Bem2 dis F and Bem2 dis R. PCR fragment (2 µg) was then transformed into individual deletion mutant. Correct integration was confirmed by diagnostic PCR and DNA sequencing of PCR fragments.

### 2.4. Construction of MPK1 Mutants

We amplified full-length *MPK1* ORF (1.4 kb) along with its native promoter (555 bp) and terminator region (130 bp) using MPK1 F and MPK1 R as forward and reverse primers from *S. cerevisiae* genomic DNA. PCR product and vector pRS313 digested with BamH1 and Xho1 were ligated and transformed in *E. coli* strain DH10B2. Transformants were screened by restriction digestion and positive clone pMPK1-313 was confirmed by automated DNA sequencing.

Three mutants of Mpk1, ATP binding site mutant (K54R), substrate binding (SB) site mutant (R196A) and D-motif binding (DB) site mutant (K83A) were made by site-directed mutagenesis using overlap extension PCR. First PCR was done to amplify 717 bp for K54R, 1.1 kb for R196A, 805 bp for K83A using MPK1 F/K54R R, MPK1 F/R196A R, and MPK1 F/K83A R primer sets, respectively. Second PCR was done to amplify 1.4 kb for K54R, 997 bp for R196A, 1.3 kb for K83A using K54R F/MPK1 R, R196A F/MPK1 R, and K83A F/MPK1 R primer sets, respectively. All the fragments were gel purified and used as the template for final overlap extension PCR to amplify 2.1 kb PCR fragments of all mutants using forward MPK1 F and reverse MPK1 R primers. All mutant constructs were then cloned in pRS313 at BamH1 and Xho1 site as described above to get pMPK1-83, pMPK1-54, and pMPK1-196. Positive clones were confirmed by automated DNA sequencing.

### 2.5. Relative Growth Assay of mpk1 Mutants

pMPK1-313 (MPK1) and its mutant constructs (pMPK1-83, pMPK1-54, and pMPK1-196) were transformed in Y00993 (*Δmpk1*) strain along with p426ClNik1. The growth pattern of these strains was checked on SD plate (-His-Ura) containing 25 µg/mL fludioxonil. For liquid media growth assay, Y00993 (*Δmpk1*) strain expressing *MPK1*, and its mutant constructs were grown overnight in SD media (-His-Ura) and then re-inoculated in the fresh media at an initial OD_600_ of 0.1. Cells were grown for four hours at 28 °C with shaking to attain log phase. The cultures were then inoculated in the 96 well plates (98 µL/well). All wells were then dispensed with serial dilutions of fludioxonil (2 µL/well). The final concentration of fludioxonil in the well was from 0–24 µg/mL. OD_600_ of the culture was measured at 0 h and after 24 h of incubation at 28 °C.

### 2.6. Western Blotting

The dually phosphorylated Hog1 in the *S. cerevisiae* strain was detected by western blotting as previously described [19]. Cells were treated with 25 µg/mL fludioxonil for various time periods ranging from 5 min to 3 h. The total cell extract (approx. 20 µg of protein) from each sample was blotted onto the nitrocellulose membrane. The dually phosphorylated Hog1 was detected using an anti-dually phosphorylated p38 antibody (Cell Signaling Technology, Danvers, MA, USA). The level of Hog1 was seen in the same blot after re-probing with anti-Hog1 antibody (Y-215, Santa Cruz Biotechnology, Dallas, TX, USA). For detecting phosphorylated Mpk1, anti-phospho-p44/42 MAPK (Thr202/Tyr204) (Rabbit) antibody was used (Cell Signaling Technology, Danvers, MA, USA).

## 3. Results

### 3.1. Identification of Fludioxonil Resistant bem2 Mutant Strain through mTn3-LacZ/LEU2 Mutagenized Library

**Figure 1:**
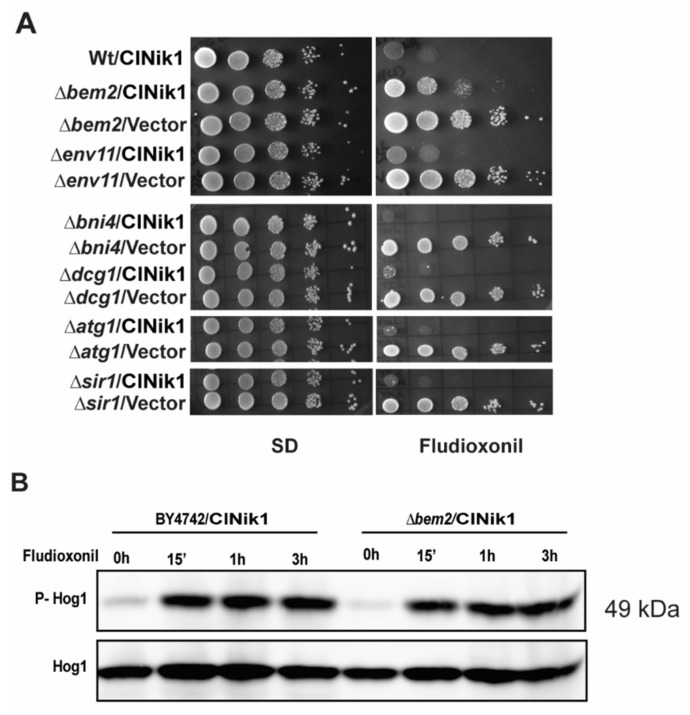
Identification of Bem2 as a downstream effector of fludioxonil toxicity. (**A**) Fludioxonil sensitivity of different *S. cerevisiae* strains identified by transposon mutagenesis by dilution spotting. Log phase cultures of different deletion mutants having pClNik1 were spotted on SD plate with or without 25 μg/mL fludioxonil and incubated for two days at 28 °C. (**B**) Western blots showing phosphorylation of Hog1 in wild type and *Δbem2* strain carrying pClNik1 upon exposure to fludioxonil. Cells were grown in SD media (-His) at 28 °C till an OD_600_~0.8 and treated with fludioxonil (25 µg/mL) at different time intervals. Blots were analyzed using an anti-phospho-p38 antibody for phospho-Hog1 (P-Hog1) and anti-Hog1 antibody for total Hog1 (Hog1).

We have utilized a *S. cerevisiae* model to conduct the genome-wide screening for fludioxonil-resistant mutants using mTn3-LacZ/LEU2 mutagenized library. We have expressed ClNik1, an HHK3 ortholog from the yeast *C. lusitaniae*, in the *S. cerevisiae* strain that conferred fludioxonil sensitivity to the host [20]. Previously, we have utilized this model to understand the antifungal activity of fludioxonil [14]. In this study, *S. cerevisiae* strain BY4742 harbouring plasmid pClNik1 [20] was transformed with mTn3-LacZ/LEU2 mutagenized library to isolate fludioxonil-resistant mutants that grew on SD-agar plate containing 50 µg/mL of fludioxonil. Out of approximately 27,000 transformants, 40 fludioxonil resistant mutants were obtained. After discarding the dominant resistant strains and the strains bearing mutations in genes of the p38/HOG MAPK pathway by genetic analysis, twelve recessive resistant strains were shortlisted for further analysis. Among these strains, the site of mTn3 insertions could be identified unambiguously only in seven resistant strains, out of which two strains had an insertion in the *BEM2* gene, and the rest showed disruption in the coding region of the following genes: *ENV11*, *BNI4*, *SIR1*, *ATG1*, and *DCG1*. To confirm that the resistance phenotype was indeed due to the deletion in the identified genes, we transformed pClNik1 into *Δbem2*, *Δenv11*, *Δbni4*, *Δsir1*, *Δatg1*, and *Δdcg1* strains obtained from EUROSCARF and checked their fludioxonil resistance (Figure 1A). We observed that only the strain carrying a deletion in *BEM2* showed resistance to fludioxonil (Figure 1A). The strain *Δenv11* showed only a partial resistance phenotype, while the other strains were sensitive to fludioxonil as was the wild type strain. Therefore, the fludioxonil resistance phenotype exhibited by *bni4*, *sir1*, *atg1*, and *dcg1* mutants obtained by transposon mutagenesis earlier could be due to the secondary site mutation unlinked to the transposon insertion site. Alternatively, the truncated protein due to transposon insertion could be responsible for the fludioxonil resistance phenotype (Supplementary Table S3). It is established that fludioxonil treatment causes the phosphorylation of Hog1 in the *S. cerevisiae* strain expressing ClNik1 [14]. Interestingly, we also observed the phosphorylation of Hog1 within 15 min of the exposure to the drug in the *Δbem2*/pClNik1 strain, and it was maintained even after a 3 h of treatment (Figure 1B). This result suggests that fludioxonil resistance exhibited by the *Δbem2* strain was not through the inactivation of the p38/HOG MAPK pathway and Bem2 might act downstream of Hog1 in fludioxonil toxicity.

**Figure 2:**
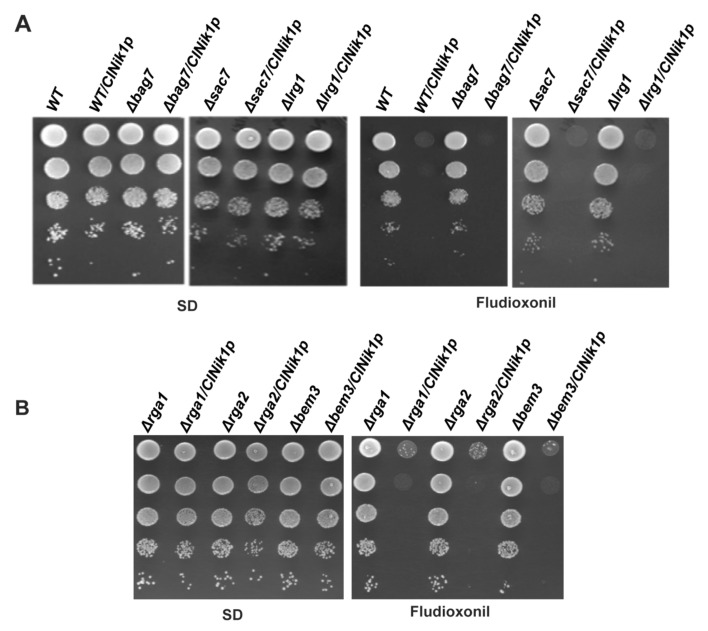
Fludioxonil sensitivity of *S. cerevisiae* strains having a deletion in different Rho1 GAP (**A**) and Cdc42 GAP (**B**) by dilution spotting. Log phase cultures of different deletion mutants having pClNik1 were spotted on SD plate with or without 25 μg/mL fludioxonil and incubated for two days at 28 °C. Representative figures from three independent experiments were shown.

Bem2 is a Rho GTPase activating protein (Rho-GAP) that acts as GAP for various GTPases Rho1, Rho2, Rho4, and Cdc42 [21,22,23,24,25,26]. Since the deletion of *BEM2* leads to fludioxonil resistance, we also checked the fludioxonil sensitivity of the deletion strains of other Rho GAPs, e.g., Y02390 (*Δbag7*), Y04225 (*Δsac7*), and Y03937 (*Δlr*g1). The expression of ClNik1 in these mutant strains conferred sensitivity to fludioxonil that was similar to that of the wild type strain (Figure 2A). In *S. cerevisiae*, Rga1, Rga2, and Bem3 are known to act as GAP for Cdc42 along with Bem2. Therefore, we also checked the fludioxonil sensitivity of the strains carrying deletion in these GAPs by dilution spotting. The strains *Δrga1*, *Δrga2*, and *Δbem3* expressing ClNik1 failed to grow on the fludioxonil plate (Figure 2B). Thus, our results indicate that the fludioxonil resistance phenotype is exclusive to the Bem2.

### 3.2. The Role of BEM2 in the Antifungal Activity of Fludioxonil

**Figure 3:**
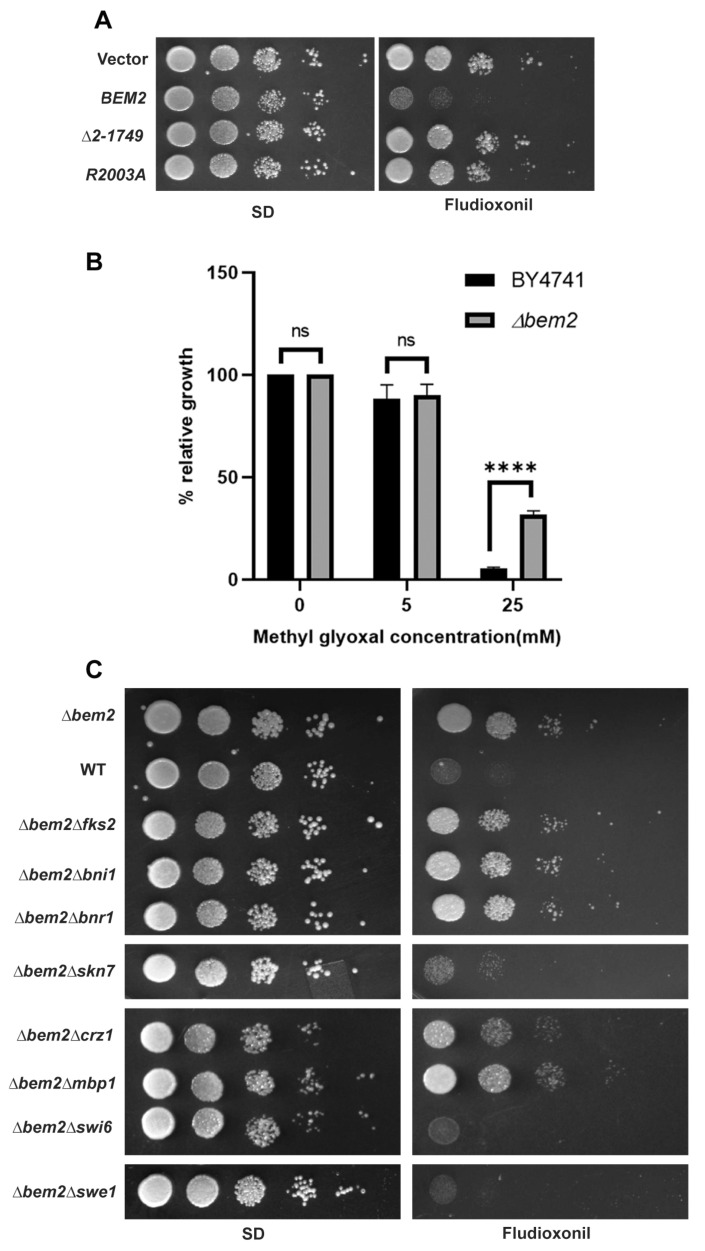
Functional analysis of fludioxonil resistance phenotype of *Δbem2* mutant.(**A**) Fludioxonil sensitivity of different *bem2* mutants by dilution spotting. Spot assays of *S. cerevisiae* strain *Δbem2* harboring plasmid pRS313 (vector), pBEM2-313 (*BEM2*), pBEM2-1749 (Δ2-1749), and pBEM2-GAP (R2003A) along with p426ClNik1 on SD or SD agar plate with 25 μg/mL fludioxonil. (**B**) Methylglyoxal sensitivity of *Δbem2* mutant by relative growth assay. Log phase culture of wild type BY4741 or *Δbem2* mutant carrying plasmid pClNik1 was inoculated in SD (-His) media with or without methylglyoxal and grown for 24 h. Relative growth is expressed as percentage of the growth (OD_600_) observed in the absence of methylglyoxal Graphs are plotted as mean ± SD. Error bars indicate standard deviations from three biological replicates assayed. Data were analyzed using Tukey’s multicomparison test. (ns, *p* > 0.05; ****, *p* < 0.0001). (**C**) Role of different downstream effectors of *BEM2*. Spot assays of *S. cerevisiae* strains *Δbem2Δfks2* (ASC 6), *Δbem2Δbni1* (ASC 4), *Δbem2Δbnr1* (ASC 5), *Δbem2Δskn7* (ASC 7), *Δbem2Δcrz1* (ASC 8), *Δbem2Δmbp1* (ASC 9), *Δbem2Δswi6* (ASC 11), and *Δbem2Δswe1* (ASC 10) harboring plasmid pClNIK1 on SD or SD agar plate with 25 μg/mL fludioxonil.

To determine whether the GAP activity or the morphogenesis checkpoint activity of Bem2 is required for fludioxonil sensitivity, we created two functional mutants of *BEM2*, R2003A and Δ2-1749. The R2003A mutant has a point mutation in the GAP domain, which completely abolishes the GAP activity of Bem2. In contrast, the Δ2-1749 mutant has a deletion in the N terminal region of Bem2, which is essential for its role in the morphogenesis checkpoint [25]. As expected, the expression of full-length *BEM2* in the *Δbem2*/p426ClNik1 strain completely reversed its resistant phenotype. However, R2003A and Δ2-1749 mutants also remained resistant to fludioxonil (Figure 3A). These results suggest that the functional GAP domain and N terminal region of Bem2 are crucial for their role in fludioxonil toxicity. Recently it has been suggested that methylglyoxal treatment phenocopies fludioxonil treatment in *S. cerevisiae* [4]. Fludioxonil treatment triggered the accumulation of cytosolic methylglyoxal in fungi, which modifies the activity of HHK3 and causes cytotoxicity. Therefore, we checked whether the deletion of *BEM2* could affect the sensitivity of the yeast towards methylglyoxal. For this, BY4741 and *Δbem2* strains carrying plasmid pClNik1 were grown in SD media or SD media supplemented with two different concentrations of methylglyoxal for 24 h. Relative growth of the strain at each concentration of methylglyoxal was expressed as a percentage of the growth (OD_600_) observed in the absence of methylglyoxal. At a lower concentration (5 mM) of methylglyoxal the relative growth of *Δbem2* and parental BY4741 was quite similar. However, at a higher concentration of methylglyoxal (25 mM), *Δbem2* showed significantly better growth than the parental strain BY4741 (Figure 3B). Thus, the deletion of *BEM2* also conferred methylglyoxal tolerance in *S. cerevisiae*.

Bem2 is a negative regulator of small GTPase Rho1, which acts as a master regulator of various cellular processes, including the CWI pathway, actin organization, and polarized secretion [27]. At the site of polarized growth, Rho1 activates various downstream effector proteins such as Fks1/Fks2, Bni1, Bnr1, Swi4, Swi6, Crz1, and Skn7. Furthermore, fludioxonil treatment also affects the cell cycle dynamics and impairs cytokinesis [14]. Therefore, we hypothesized that if Bem2 acts upstream of these effector proteins by regulating Rho1 activity and its morphogenesis checkpoint role, then the deletion of the downstream effector proteins in the *BEM2* deletion background will affect its sensitivity towards fludioxonil. We shortlisted a set of genes involved in *BEM2* controlled cellular processes and checked the fludioxonil sensitivity of all the double deletion strains expressing ClNik1. We observed that the deletion of *SWE1* and *SWI6* suppressed the resistance of *Δbem2* completely (Figure 3C), while the deletion of *BEM2* in *mpk1* and *swi4* background could not be made as they appear to be lethal to the cells. Synthetic lethality between *bem2* and *swi4* mutants has been reported previously [28]. Interestingly, the deletion of *SKN7* suppressed the resistance partially. In contrast, the deletion of *BNI1*, *BNR1*, *CRZ1*, *FKS2*, and *MBP1* in the *BEM2* deletion background did not affect the drug resistance phenotype (Figure 3C). These results indicate that the genes involved in the CWI pathway and cell cycle regulation contribute to the viability of *Δbem2* cells in response to fludioxonil.

### 3.3. The Activation of CWI Pathway upon Fludioxonil Treatment

**Figure 4:**
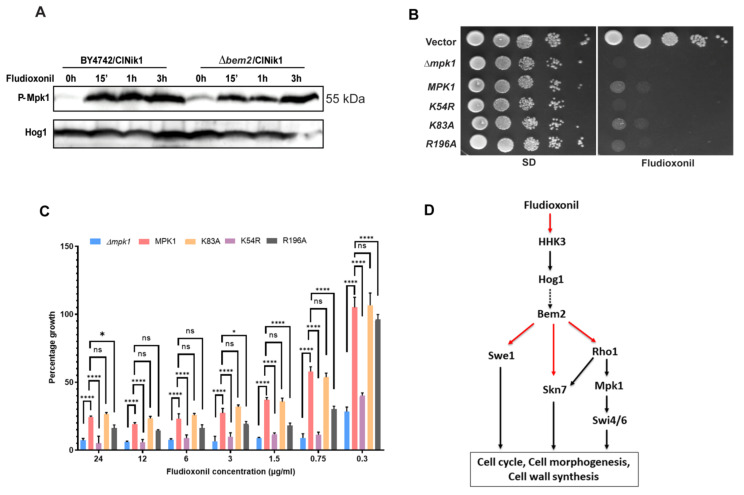
Role of CWI pathway in the cytotoxic effect of fludioxonil in *S. cerevisiae.* (**A**) Western blotting showing the activation of CWI pathway upon fludioxonil treatment. Cells were grown in SD media (-His) at 28 °C until an OD_600_ of ~0.8 and treated with fludioxonil (25 µg/mL) for different time intervals. Total cell extract from these samples was used for western blotting analysis. Blots were analyzed using an anti-phospho-p44/42 MAPK antibody that recognizes the dual-phosphorylated form of ScMpk1 and anti-Hog1 antibody for total Hog1 (Hog1) for loading control. The data shown are representative of three independent experiments. (**B**) Fludioxonil sensitivity of Δ*mpk1* strain harboring two plasmids pRS313 and p426ClNik1 (Δ*mpk1*), pMPK1-313 and p426ClNik1 (MPK1), pMPK1-54 and p426ClNik1 (K54R), pMPK1-83 and p426ClNik1 (K83A), and pMPK1-196 and p426Nik1 (R196A) by dilution spotting on SD plate with 25 μg/mL fludioxonil. Δ*mpk1* strain harboring plasmids pRS313 and pRS426 (vector) was used as control. The data shown are representative of three independent experiments. (**C**) Relative growth of *Δmpk1* strain harboring plasmids pRS313 (*Δmpk1*), pMPK1-313 (MPK1), pMPK1-83 (K83A), pMPK1-54 (K54R), pMPK1-196 (R196A) along with p426ClNIK1 on SD liquid media containing different concentrations of fludioxonil. The log phase culture of each mutant was used to inoculate the 96 well plates (98 µL/well) having serial dilutions of fludioxonil (2 µL/well). OD_600_ of each well at 0 h and then after 24 h of incubation at 28 °C was recorded. The graph represents the relative growth of the culture at each concentration of fludioxonil that was expressed as the percentage of the growth measured in the absence of fludioxonil. Graphs are plotted as mean ± SD. Error bars indicate standard deviations from three biological replicates assayed. Data were analyzed using Tukey’s multicomparison test. (ns, *p* > 0.05; *, *p* < 0.05; ****, *p* < 0.0001). (**D**) Schematic representation of the role of Bem2 and its downstream target genes in the antifungal action of fludioxonil. Red arrows: negative interaction between the genes and the drug. Dotted arrows: genetic interaction unknown. Black arrows: positive interaction between the genes.

Bem2 modulates the activity of Rho1, which is a master regulator CWI pathway in yeast. Previous studies showed that the CWI pathway plays a very important role in the antifungal action of fludioxonil [29,30]. The *Δmpk1* MAPK mutant strain of *C. neoformans* exhibited fludioxonil hypersensitivity. Moreover, in *Aspergillus fumigatus*, cell wall composition was affected by fludioxonil treatment [30]. To further explore the role of the CWI pathway, we checked the levels of dually phosphorylated Mpk1 using immunoblotting (Figure 4A). Fludioxonil treatment caused Mpk1 phosphorylation within 15 min and was maintained up to 3 h in both BY4742 and *Δbem2* strains. Interestingly, the basal level of phosphorylated Mpk1 was seen even in the absence of the drug in the *Δbem2* strain, confirming the over activation of Mpk1 in this genetic background. Thus, fludioxonil activates the CWI pathway in the *S. cerevisiae* strain expressing ClNik1.

Mpk1 phosphorylation regulates the expression of various genes through further activation of two distinct transcription factors, SBF (Swi4/Swi6) and Rlm1 [31,32,33,34]. Mpk1 binds to its substrates and regulators through two distant interacting sites, the D motif binding site (DB) and substrate binding site (SB). Mpk1 binding to Swi4 requires its intact DB site but not the SB site; however, the SB site seems crucial for Rlm1-driven transcription [35]. We generated three mutants of Mpk1, ATP binding site mutant (K54R), substrate binding (SB) site mutant (R196A), and D-motif binding (DB) site mutant (K83A), by site-directed mutagenesis. The growth pattern of the mutant strains expressing ClNik1 was checked on fludioxonil agar media. We observed that the full-length *MPK1* and DB site mutant K83A alleviated the hypersensitivity of the *Δmpk1* strain towards fludioxonil. On the other hand, the ATP binding site mutant K54R behaved as a non-functional kinase and showed hypersensitivity to the drug. Interestingly, the SB site mutant R196A showed lesser growth than full-length *MPK1* (Figure 4B). To better understand the growth pattern, we also checked the growth of these strains in the presence of serial concentrations of fludioxonil (0–24 µg/mL) in liquid media. We observed the same pattern, as R196A mutant showed a significant two-fold decrease in growth compared to *MPK1* at 0.75 µg/mL fludioxonil, which indicated that Rlm1 binding to the SB site was crucial for Mpk1 function in fludioxonil mechanism of action (Figure 4C). Thus, our results confirm that cell wall integrity plays a central role in the fludioxonil tolerance.

## 4. Discussion

Fludioxonil toxicity is not solely the outcome of increased osmotic pressure, but other cellular processes, i.e., cytokinesis, cell trafficking, and cell wall integrity, are compromised, which contributes to the growth arrest and cell death [14,30]. This study takes a step further to identify genes or pathways involved in the fludioxonil mode of action. We performed insertion mutagenesis to screen for the strains resistant to fludioxonil. Our screening identified *BEM2* as a potential regulator of fludioxonil toxicity. Bem2 encodes a Rho GTPase activating protein that regulates GTPases to maintain morphogenesis and cell polarity in *S. cerevisiae* [25]. *BEM2* deletion leads to various cellular defects, which includes an actin polarity defect, a septin assembly defect, a cytokinesis defect, hyper-activation of CWI pathway, enlarged vacuoles, and a morphogenesis checkpoint defect [23,25,36,37,38,39,40]. Bem2 acts as GAP proteins for Rho1 and Cdc42 [27]. Apart from Bem2, Sac1, Lrg1, and Bag7 also function as GAP for Rho1 protein in *S. cerevisiae*. Similarly, Cdc42 is also regulated by other GAP proteins, e.g., Rga1, Rga2, and Bem3. Our results showed that the fludioxonil sensitivity of the cell was not affected by the deletion of anyone of these GAPs (Figure 2). Thus, Bem2 is the only Rho GAP that seems to have a role in fludioxonil toxicity.

Moreover, the dually phosphorylated Hog1 was detected within fifteen minutes of fludioxonil treatment in the *Δbem2*/pClNik1 strain (Figure 1B), which ruled out the possibility that fludioxonil resistance exhibited by the *Δbem2* strain was due to the inactivation of the p38/HOG MAPK pathway. This observation suggests that Bem2 is either acting downstream of Hog1 or the fludioxonil resistance of *Δbem2* strain is entirely an independent phenomenon. To delineate the role of Bem2 in fludioxonil sensitivity, we constructed two functional mutants, R2003A and Δ2-1749, which abolish the GAP activity and morphogenesis checkpoint role, respectively. The expression of full-length *BEM2* restored the fludioxonil sensitivity to the *Δbem2*/pClNik1 strain. However, both the mutant constructs were still resistant to fludioxonil, indicating the role of both the GAP domain and the N terminal region of Bem2 in conferring fludioxonil sensitivity.

Furthermore, we performed a genetic interaction study to determine how the downstream effector of Bem2 contributed to the drug resistance phenotype. Our results confirmed that Swi6, Swe1, and Skn7 play a crucial role in the resistance phenotype of the *Δbem2*/pClNik1 strain. Since *swi4* exhibit synthetic lethal interactions with *bem2*, its effect on the fludioxonil resistance phenotype could not be tested directly. Swi6 and Swi4 directly contribute to CWI transcriptional program whereas Swe1 is required for the morphogenesis checkpoint [41]. This checkpoint delays the cell cycle at the G2 phase in response to stresses that perturb the actin cytoskeleton and septin or bud formation. Our results indicate that the morphogenesis checkpoint role of *BEM2* is also crucial for drug-mediated activity (Figure 3A). Interestingly, the CWI pathway also plays an important role in the morphogenesis checkpoint. Skn7 is regulated by both the p38/HOG MAPK pathway and Rho1GTPase and has a role in cell wall synthesis [10,27]. The role of *SKN7* in the fungicidal action of fludioxonil has also been presented recently [11,30]. Our results indicate that the deletion of *SKN7* in the *bem2* strain might reduce cell wall rigidity; hence, cells become more sensitive to the drug.

The hypersensitivity of *mpk1* mutants and the overrepresentation of cell wall genes in the deletion sensitivity profiling (DSP) assay of fludioxonil indicate that the CWI pathway contributes to the resistance to the drug [14]. The CWI pathway regulates the new cell wall synthesis, which is pivotal for budding and polarized growth in fungi. An early study in *C. neoformans* revealed that the deletion of *MPK1* leads to fludioxonil hypersensitivity [29], and similar results were observed in the *S. cerevisiae* strain expressing ClNik1 [14,42]. We have observed Mpk1 phosphorylation within 15 min of fludioxonil treatment in both BY4742 and *Δbem2* strains (Figure 4A). Bem2 is a negative regulator of the CWI pathway, and its deletion leads to constitutive Mpk1 activation [43,44]. The basal level of phosphorylated Mpk1 was observed even under normal conditions in the *BEM2* deletion strain. The role of Mpk1 in fludioxonil sensitivity was further explored by creating a set of mutations: K83A, R196A, and K54R, which abolished the binding of proteins at the DB site, the SB site, and ATP binding, respectively. The SB site mutant R196A, which was defective in transcription through Rlm1, showed the two-fold decrease in growth compared to MPK1, suggesting the role of Rlm1-driven transcription of cell wall genes in providing resistance to the cell on fludioxonil treatment (Figure 4C). All these observations indicated the role of cell wall integrity and morphogenesis checkpoint control in maintaining cell viability during fludioxonil treatment. Various environmental and integral cellular factors also trigger the activation of the CWI pathway thatfurther regulates cell cycle, morphogenesis checkpoint, and septum dynamics [45], and Bem2 also has a role in these processes. Summing up, our work provides a novel chemical genetic link between Bem2 and the cell wall integrity pathway. We speculate that in the presence of fludioxonil, Bem2 negatively regulates the cell wall integrity pathway kinase Mpk1, and the transcription factor Skn7 through Rho1 GTPase, as depicted schematically in Figure 4D. In future, deciphering this at the molecular level would provide a novel insight into the fungicidal action of fludioxonil.

## Figures and Tables

**Table 1:** List of Primers used in this study.

Primer	Sequence
For cloning *BEM2* and its mutants	
Bem2 PF	CGCTCGAGCGAGACGAAGAGCTGAGCACCAAGAG
Bem2 Kas R	GGCGCCGATTTGGATTTTTGCCATA
Bem2 Kas F	GGCGCCGCGGAAGCTATACTACATT
Bem2 ORF R	CGAGCTCGTTATTGCTTGAAATAATCATTTGGATTCT
Bem2 Sph1 F	GCATGCCATAATATCACCAGAGTCTAG
R2003A F	GTGGGATTGTACGCTATTCCTGGTTCCATCGG
R2003A R	GATGGAACCAGGAATAGCGTACAATCCCACTTCATCT
Δ2-1749 F	AGGAGTCTAATGATCTTGAAGAACTCTGCTGCTTTAC
Δ2-1749 R	AGAGTTCTTCAAGATCATTAGACTCCTGCTTCGTTATTT
For disruption of *BEM2*	
Bem2 dis F	CTGGATAGACACAAAAAAAACAAATAACGAAGCAGGAGTCTAATGAAAGGCAGCTGAAGCTTCGTACGC
Bem2 dis R	CTCTCTCAGCAGTGGATTGTATACATTTACCACGAAAATTGTTTATTGCTGCATAGGCCACTAGTGGATCTG
Bem2 conf F	CACTGGTACTGTCTGCTAACTCAAA
Bem2 conf R	AAACATAACATTCAAAAGGCAAGAG
Lacz F	GAGGTCGACGGTATCGATAAGC
Lacz R	CCCAGTCACGACGTTGTAAAAC
For cloning *MPK1* and its mutants	
MPK1 F	CCCTCGAGGGCGGTAACTATGGACACCTTACAGG
MPK1 R	CGGGATCCCGACGAGCTACAACAAGAGCACGTA
K54R F	CAGTTGCCATCAGAAAAGTGACAAACGTTTTTTCGA
K54R R	GTTTGTCACTTTTCTGATGGCAACTGTGGTATCTTCG
K83A F	TCAGAGGCCACGCTAATATTACATGTCTTTATGATATGGATATTG
K83A R	CATGTAATATTAGCGTGGCCTCTGAAATGTCTCAAA
R196A F	ACGTGGCCACTGCTTGGTATAGAGCTCCGGAAATAATG
R196A R	5 GCTCTATACCAAGCAGTGGCCACGTACTCCGTC

## Data Availability

All data generated or analyzed during this study are included within the article.

## Supplementary Materials

The following are available online at https://www.mdpi.com/article/10.3390/jof8070754/s1, Table S1: List of strains used in this study; Table S2: List of plasmids used in this study [46,47]; Table S3: Details of transposon insertion sites.

## References

[1] Kilani J., Fillinger S. (2016). Phenylpyrroles: 30 years, two molecules and (nearly) no resistance. Front. Microbiol..

[2] Tanaka T., Izumitsu K., Carisse O. (2010). Two-Component signaling system in filamentous fungi and the mode of action of dicarboximide and phenylpyrrole fungicides. Fungicides.

[3] Bersching K., Jacob S. (2021). The Molecular Mechanism of Fludioxonil Action Is Different to Osmotic Stress Sensing. J. Fungi.

[4] Brandhorst T.T., Kean I.R.L., Lawry S.M., Wiesner D.L., Klein B.S. (2019). Phenylpyrrole fungicides act on triosephosphate isomerase to induce methylglyoxal stress and alter hybrid histidine kinase activity. Sci. Res..

[5] Cui W., Beever R.E., Parkes S.L., Weeds P.L., Templeton M.D. (2002). An osmosensing histidine kinase mediates dicarboximide fungicide resistance in *Botryotinia fuckeliana* (*Botrytis cinerea*). Fungal Genet. Biol..

[6] Fujimura M., Ochiai N., Ichiishi A., Usami R., Horikoshi K., Yamaguchi I. (2000). Fungicide resistance and osmotic stress sensitivity in *os* mutants of *Neurospora crassa*. Pestic. Biochem. Phys..

[7] Motoyama T., Ohira T., Kadokura K., Ichiishi A., Fujimura M., Yamaguchi I., Kudo T. (2005). An Os-1 family histidine kinase from a filamentous fungus confers fungicide-sensitivity to yeast. Curr. Genet..

[8] Yoshimi A., Tsuda M., Tanaka C. (2004). Cloning and characterization of the histidine kinase gene Dic1 from *Cochliobolus heterostrophus* that confers dicarboximide resistance and osmotic adaptation. Mol. Genet. Genom..

[9] Zhang Y., Lamm R., Pillonel C., Lam S., Xu J.R. (2002). Osmoregulation and fungicide resistance: The *Neurospora crassa* os-2 gene encodes a HOG1 mitogen-activated protein kinase homologue. Appl. Environ. Microbiol..

[10] Izumitsu K., Yoshimi A., Hamada S., Morita A., Saitoh Y., Tanaka C. (2009). Dic2 and Dic3 loci confer osmotic adaptation and fungicidal sensitivity independent of the HOG pathway in *Cochliobolus heterostrophus*. Mycol. Res..

[11] Bahn Y.S., Kojima K., Cox G.M., Heitman J. (2006). A unique fungal two-component system regulates stress responses, drug sensitivity, sexual development, and virulence of *Cryptococcus neoformans*. Mol. Biol. Cell.

[12] Buschart A., Gremmer K., El-Mowafy M., van den Heuvel J., Mueller P.P., Bilitewski U. (2012). A novel functional assay for fungal histidine kinases group III reveals the role of HAMP domains for fungicide sensitivity. J. Biotechnol..

[13] Furukawa K., Randhawa A., Kaur H., Mondal A.K., Hohmann S. (2012). Fungal fludioxonil sensitivity is diminished by a constitutively active form of the group III histidine kinase. FEBS Lett..

[14] Randhawa A., Kundu D., Sharma A., Prasad R., Mondal A.K. (2019). Overexpression of the CORVET complex alleviates the fungicidal effects of fludioxonil on the yeast *Saccharomyces cerevisiae* expressing hybrid histidine kinase 3. J. Biol. Chem..

[15] Gietz R.D., Woods R.A. (2002). Transformation of yeast by lithium acetate/single-stranded carrier DNA/polyethylene glycol method. Methods Enzymol..

[16] Burns N., Grimwade B., Ross-Macdonald P.B., Choi E.Y., Finberg K., Roeder G.S., Snyder M. (1994). Large-scale analysis of gene expression, protein localization, and gene disruption in *Saccharomyces cerevisiae*. Genes Dev..

[17] Sikorski R.S., Hieter P. (1989). A system of shuttle vectors and yeast host strains designed for efficient manipulation of DNA in *Saccharomyces cerevisiae*. Genetics.

[18] Gueldener U., Heinisch J., Koehler G.J., Voss D., Hegemann J.H. (2002). A second set of loxP marker cassettes for Cre-mediated multiple gene knockouts in budding yeast. Nucleic Acids Res..

[19] Meena N., Kaur H., Mondal A.K. (2010). Interactions among HAMP Domain repeats act as an osmosensing molecular switch in group III hybrid histidine kinases from fungi. J. Biol. Chem..

[20] Randhawa A., Chawla S., Mondal A.K. (2016). Functional dissection of HAMP domains in NIK1 ortholog from pathogenic yeast *Candida lusitaniae*. Gene.

[21] Bender A., Pringle J.R. (1989). Multicopy suppression of the *cdc24* budding defect in yeast by *CDC42* and three newly identified genes including the ras-related gene RSR1. Proc. Natl. Acad. Sci. USA.

[22] Gong T., Liao Y., He F., Yang Y., Yang D.D., Chen X.D., Gao X.D. (2013). Control of polarized growth by the Rho family GTPase Rho4 in budding yeast: Requirement of the N-terminal extension of Rho4 and regulation by the Rho GTPase-activating protein Bem2. Eukaryot. Cell.

[23] Kim Y.J., Francisco L., Chen G.C., Marcotte E., Chan C.S. (1994). Control of cellular morphogenesis by the Ip12/Bem2 GTPase-activating protein: Possible role of protein phosphorylation. J. Cell Biol..

[24] Knaus M., Pelli-Gulli M.P., van Drogen F., Springer S., Jaquenoud M., Peter M. (2007). Phosphorylation of Bem2p and Bem3p may contribute to local activation of Cdc42p at bud emergence. EMBO J..

[25] Marquitz A.R., Harrison J.C., Bose I., Zyla T.R., McMillan J.N., Lew D.J. (2002). The Rho-GAP Bem2p plays a GAP-independent role in the morphogenesis checkpoint. EMBO J..

[26] Peterson J., Zheng Y., Bender L., Myers A., Cerione R., Bender A. (1994). Interactions between the bud emergence proteins Bem1p and Bem2p and Rho-type GTPases in yeast. J. Cell Biol..

[27] Levin D.E. (2011). Regulation of cell wall biogenesis in *Saccharomyces cerevisiae*: The cell wall integrity signaling pathway. Genetics.

[28] Jorgensen P., Nishikawa J.L., Breitkreutz B.J., Tyers M. (2002). Systematic identification of pathways that couple cell growth and division in yeast. Science.

[29] Kojima K., Bahn Y.S., Heitman J. (2006). Calcineurin, Mpk1 and Hog1 MAPK pathways independently control fludioxonil antifungal sensitivity in *Cryptococcus neoformans*. Microbiology.

[30] Schruefer S., Bohmer I., Dichtl K., Spadinger A., Kleinemeier C., Ebel F. (2021). The response regulator Skn7 of *Aspergillus fumigatus* is essential for the antifungal effect of fludioxonil. Sci. Rep..

[31] Baetz K., Moffat J., Haynes J., Chang M., Andrews B. (2001). Transcriptional co-regulation by the cell integrity mitogen-activated protein kinase Slt2 and the cell cycle regulator Swi4. Mol. Cell. Biol..

[32] Dodou E., Treisman R. (1997). The *Saccharomyces cerevisiae* MADS-box transcription factor Rlm1 is a target for the Mpk1 mitogen-activated protein kinase pathway. Mol. Cell. Biol..

[33] Madden K., Sheu Y.J., Baetz K., Andrews B., Snyder M. (1997). SBF cell cycle regulator as a target of the yeast PKC-MAP kinase pathway. Science.

[34] Watanabe Y., Takaesu G., Hagiwara M., Irie K., Matsumoto K. (1997). Characterization of a serum response factor-like protein in *Saccharomyces cerevisiae*, Rlm1, which has transcriptional activity regulated by the Mpk1 (Slt2) mitogen-activated protein kinase pathway. Mol. Cell. Biol..

[35] Truman A.W., Kim K.Y., Levin D.E. (2009). Mechanism of Mpk1 mitogen-activated protein kinase binding to the Swi4 transcription factor and its regulation by a novel caffeine-induced phosphorylation. Mol. Cell. Biol..

[36] Atkins B.D., Yoshida S., Saito K., Wu C.F., Lew D.J., Pellman D. (2013). Inhibition of Cdc42 during mitotic exit is required for cytokinesis. J. Cell Biol..

[37] Cid V.C.J., Adamikova L., Sanchez M., Molina M.A., Nombela C. (2001). Cell cycle control of septin ring dynamics in the budding yeast. Microbiology.

[38] Cid V.J., Cenamor R., Sanchez M., Nombela C. (1998). A mutation in the Rho1-GAP-encoding gene BEM2 of *Saccharomyces cerevisiae* affects morphogenesis and cell wall functionality. Microbiology.

[39] Seeley E.S., Kato M., Margolis N., Wickner W., Eitzen G. (2002). Genomic analysis of homotypic vacuole fusion. Mol. Biol. Cell.

[40] Wang T., Bretscher A. (1995). The rho-GAP encoded by BEM2 regulates cytoskeletal structure in budding yeast. Mol. Biol. Cell.

[41] Sia R.A., Bardes E.S., Lew D.J. (1998). Control of Swe1p degradation by the morphogenesis checkpoint. EMBO J..

[42] Randhawa A., Mondal A.K. (2013). The sixth HAMP domain negatively regulates the activity of the group III HHK containing seven HAMP domains. Biochem. Biophys. Res. Commun..

[43] Borah S., Shivarathri R., Kaur R. (2001). The Rho1 GTPase-activating protein CgBem2 is required for survival of azole stress in *Candida glabrata*. J. Biol. Chem..

[44] Martin H., Rodriguez-Pachon J.M., Ruiz C., Nombela C., Molina M. (2000). Regulatory mechanisms for modulation of signaling through the cell integrity Slt2-mediated pathway in *Saccharomyces cerevisiae*. J. Biol. Chem..

[45] Roncero C., Celador R., Sánchez N., García P., Sánchez Y. (2021). The Role of the Cell Integrity Pathway in Septum Assembly in Yeast. J. Fungi.

[46] Mumberg D., Muller R., Funk M. (1995). Yeast vectors for the controlled expression of heterologous proteins in different genetic backgrounds. Gene.

[47] Christianson T.W., Sikorski R.S., Dante M., Shero J.H., Hieter P. (1992). Multifunctional yeast high-copy-number shuttle vectors. Gene.

