# Supplementary material for: BEM2, a RHO GTPase Activating Protein That Regulates Morphogenesis in S. cerevisiae, Is a Downstream Effector of Fungicidal Action of Fludioxonil
Source: J Fungi (Basel). 2022 Jul 21;8(7):754. doi: 10.3390/jof8070754 (PMC9316689; doi:10.3390/jof8070754)
Supplement: Supplementary file 1 [file jof-08-00754-s001.zip › jof-1765706-supplementary.pdf]

Supplementary data

Table S1. List of strains used in this study.

Strains	Organism	Genotype	Reference
**DH 10 B**	*E. coli*	F- *end A1 recA1 galE15 galK16 nupGrpsLΔlacX74 (p80lac ZΔM15 araD139 Δ(ara,leu)7697 mcrAΔ (mrrhsdRMS-mcrBC)λ*	Lab strain
**BY4741**	*S. cerevisiae*	*MATa ura3Δ0 leu2Δ0 his3Δ1 met2Δ0*	Lab strain
**BY4742**	*S. cerevisiae*	*MATa; his3Δ1; leu2Δ0; lys2Δ0; ura3Δ0*	Lab strain
**Y16152**	*S. cerevisiae*	BY4742;*MATa his3Δ1; leu2Δ0; lys2Δ0; ura3Δ0,BEM2*::KanMX4	Euroscarf
**Y14701**	*S. cerevisiae*	BY4742;*MATa his3Δ1; leu2Δ0; lys2Δ0; ura3Δ0,ENV11*::KanMX4	Euroscarf
**Y11979**	*S. cerevisiae*	BY4742;*MATa his3Δ1; leu2Δ0; lys2Δ0; ura3Δ0,BNI4*::KanMX4	Euroscarf
**Y17304**	*S. cerevisiae*	BY4742;*MATa his3Δ1; leu2Δ0; lys2Δ0; ura3Δ0,DCG1*::KanMX4	Euroscarf
**Y14547**	*S. cerevisiae*	BY4742;*MATa his3Δ1 ; leu2Δ0; lys2Δ0; ura3Δ0,ATG1*::KanMX4	Euroscarf
**Y16017**	*S. cerevisiae*	BY4742;*MATa his3Δ1; leu2Δ0; lys2Δ0; ura3Δ0,SIR1*::KanMX4	Euroscarf
**Y03937**	*S. cerevisiae*	BY4741;*MATa ura3Δ0 leu2Δ0 his3Δ1 met2Δ0, LRG1*::KanMX4	Euroscarf
**Y02390**	*S. cerevisiae*	BY4741;*MATa ura3Δ0 leu2Δ0 his3Δ1 met2Δ0, BAG7*::KanMX4	Euroscarf
**Y04225**	*S. cerevisiae*	BY4741;*MATa ura3Δ0 leu2Δ0 his3Δ1 met2Δ0, SAC7*::KanMX4	Euroscarf
**Y00993**	*S. cerevisiae*	BY4741;*MATa ura3Δ0 leu2Δ0 his3Δ1 met2Δ0, MPK1*::KanMX4	Euroscarf
**Y02383**	*S. cerevisiae*	BY4741;*MATa ura3Δ0 leu2Δ0 his3Δ1 met2Δ0, RGA1*::KanMX4	Euroscarf
**Y04215**	*S. cerevisiae*	BY4741;*MATa ura3Δ0 leu2Δ0 his3Δ1 met2Δ0, RGA2*::KanMX4	Euroscarf
**Y02137**	*S. cerevisiae*	BY4741;*MATa ura3Δ0 leu2Δ0 his3Δ1 met2Δ0, BEM3*::KanMX4	Euroscarf
**YPDahl143**	*S. cerevisiae*	*MATa leu23/112, ura3Δ0,trp1-1,his3Δ15 ade21,can1100 GAL SUC2 SSK1::KanMX, SHO::TRP1*	Lab strain

**ASC 4**	*S. cerevisiae*	BY4741; *BNI1*::*KanMX4,BEM2*::*URA3*	Present study
**ASC 5**	*S. cerevisiae*	BY4741; *BNR1*::*KanMX4*, *BEM2*::*URA3*	Present study
**ASC 6**	*S. cerevisiae*	BY4741; *FKS2*::*KanMX4*, *BEM2*::*URA3*	Present study
**ASC 7**	*S. cerevisiae*	BY4741;*SKN7*::*KanMX4,BEM2*::*URA3*	Present study
**ASC 8**	*S. cerevisiae*	BY4741;*CRZ1*::*KanMX4,BEM2*::*LEU2*	Present study
**ASC 9**	*S. cerevisiae*	BY4741;*MBP1*::*KanMX4, BEM2*::*LEU2*	Present study
**ASC 10**	*S. cerevisiae*	BY4741;*SWE1*::*KanMX4,BEM2*::*LEU2*	Present study
**ASC 11**	*S. cerevisiae*	BY4741;*SWI6*::*KanMX4, BEM2*::*LEU2*	Present study

Table S2. List of plasmids used in this study.

Plasmids	Description	Source
p423 TEF	*HIS3 2μ*	Mumberg et al. [46]
p426 TEF	*URA3 2μ*	Mumberg et al. [46]
pGEM7Z	Amp^r *lacZ* polylinker	Promega
pRS423	*HIS3 2μ*	Christianson et al. [47]
pRS313	*HIS3 ARS*	Sikorski et al. [17]
pClNik1	*ClNIK1* ORF cloned in p423 TEF	Randhwa et al. [20]
p426-ClNik1	*ClNIK1* ORF cloned in p426 TEF	Randhwa et al. [20]
pUG 72	*loxP-URA3-loxP*	Gueldener et al. [18]
pUG 73	*loxP-LEU2-loxP*	Gueldener et al. [18]
pBEM2-313	*BEM2* ORF cloned in pRS313	Present Study
pBEM2-GAP	R2003A mutant of *BEM2* cloned in pRS313	Present Study
pBEM2-1749	Δ2-1749 mutant of *BEM2* cloned in pRS313	Present Study
pMPK1-313	*MPK1* ORF cloned in pRS313	Present Study
pMPK1-83	K83A mutant of *MPK1* cloned in pRS313	Present Study
pMPK1-54	K54R mutant of *MPK1* cloned in pRS313	Present Study
pMPK1-196	R196A mutant of *MPK1* cloned in pRS313	Present Study

Table S3. Details of transposon insertion sites.

Genes	ORF size (bp)	Transposon insertion site in ORF (bp)
BEM2	**6504**	**3807**
ENV11	**2583**	**1692**
BNI4	**2679**	**802**
DCG1	**735**	**478**
ATG1	**2694**	**823**
SIR1	**1965**	**627**
